# Computational Analysis of Polymeric Biodegradable and Customizable Airway Stent Designs

**DOI:** 10.3390/polym16121691

**Published:** 2024-06-14

**Authors:** Ada Ayechu-Abendaño, Aurora Pérez-Jiménez, Carmen Sánchez-Matás, José Luis López-Villalobos, Cristina Díaz-Jiménez, Rocío Fernández-Parra, Mauro Malvè

**Affiliations:** 1 Department of Engineering, Public University of Navarra (UPNA), Campus Arrosadía, s/n, E-31006 Pamplona, Spain; ayechu.120935@e.unavarra.es (A.A.-A.); aperez@ain.es (A.P.-J.); 2 AIN—Asociación de la Industria Navarra, Ctra. Pamplona, 1. Edif. AIN, E-31191 Cordovilla, Spain; cdiaz@ain.es; 3 Department of Thoracic Surgery, University Hospital Virgen de la Arrixaca, Ctra. Madrid-Cartagena, s/n, E-30120 El Palmar, Spain; nem.csm@gmail.com; 4 Department of Thoracic Surgery, University Hospital Virgen del Rocío, Avenida Manuel Siurot, s/n, E-41013 Sevilla, Spain; jllopezvillalobos@gmail.com; 5 Department of Small Animal Medicine and Surgery, Faculty of Veterinary Medicine, Catholic University of Valencia San Vicente Mártir, E-46001 Valencia, Spain; rocio.f.parra@ucv.es; 6 Research Networking in Bioengineering, Biomaterials & Nanomedicine (CIBER-BBN), Av. Monforte de Lemos, 3-5, Pabellón 11, Planta 0, E-28029 Madrid, Spain

**Keywords:** biodegradable airway stent, polylactic acid, finite element method, parametric model, 3D printing, customized prosthesis

## Abstract

The placement of endotracheal prostheses is a procedure used to treat tracheal lesions when no other surgical options are available. Unfortunately, this technique remains controversial. Both silicon and metallic stents are used with unpredictable success rates, as they have advantages but also disadvantages. Typical side effects include restenosis due to epithelial hyperplasia, obstruction and granuloma formation. Repeat interventions are often required. Biodegradable stents are promising in the field of cardiovascular biomechanics but are not yet approved for use in the respiratory system. The aim of the present study is to summarize important information and to evaluate the role of different geometrical features for the fabrication of a new tracheo-bronchial prosthesis prototype, which should be biodegradable, adaptable to the patient’s lesion and producible by 3D printing. A parametric design and subsequent computational analysis using the finite element method is carried out. Two different stent designs are parameterized and analyzed. The biodegradable material chosen for simulations is polylactic acid. Experimental tests are conducted for assessing its mechanical properties. The role of the key design parameters on the radial force of the biodegradable prosthesis is investigated. The computational results allow us to elucidate the role of the pitch angle, the wire thickness and the number of cells or units, among other parameters, on the radial force. This work may be useful for the design of ad hoc airway stents according to the patient and type of lesion.

## 1. Introduction

Tracheal and bronchial stenoses are constrictions of the airway lumen that can lead to death if not treated in time and adequately [1,2]. This pathology can be congenital, caused by the collapse or degeneration of the cartilaginous rings and the muscular membrane, or acquired, due to infection, inflammation, trauma, tracheomalacia, fibrous stenosis and benign or malignant tumors [3,4]. There are several clinical treatments currently available to treat these pathologies. However, there is no specific treatment for each situation, and in general, there are several associated morbidities [3,5,6,7]. Among the various options, when surgical treatment is not possible, an airway stent (also called a tracheobronchial prosthesis or endoprosthesis) is often placed by bronchoscopy as a last resort. Depending on the country and experience, silicone or metal stents are used. Unfortunately, in the vast majority of cases, the insertion of a silicone prosthesis requires a new procedure to replace a migrated device or to remove mucus [8]. In general, migration, inflammation and obstruction have been frequently reported as postoperative complications of silicone prostheses [8,9]. The placement of a covered metallic stent is another effective way to restore the tracheal lumen in case of stenosis [10,11]. Many studies have reported good results for both benign and malignant pathologies in the treatment of central lesions in the human airway [12,13,14]. The introduction of self-expanding metallic stents is indicated for malignant pathologies. On the contrary, the use of these devices in benign pathologies is unclear. However, the Food and Drug Administration (FDA) recommends the use of a metallic stent for benign pathologies only when no other means such as surgery or insertion of silicone stents are possible [15,16,17].

Ideally, a tracheobronchial prosthesis should meet several requirements: it should be biocompatible, impermeable, flexible and sufficiently stiff in the radial direction to allow physiological maneuvers and prevent collapse, removable and easy to place or replace, and inexpensive. [1,18]. Considering the obvious difficulties to meet all these requirements simultaneously, poor clinical results are often obtained also due to the lack of personalization in the devices [19]. The commercially available prostheses are currently offered in different sizes, but the personalization is not efficient as it only concerns the diameters and lengths of the prosthesis, leaving the main design unchanged [20]. For this reason, in recent years, three-dimensional (3D) printing has started interesting clinical environments. Rapid prototyping and fabrication of patient-specific anatomical shapes and medical devices is not only feasible but also directly applicable to patients, as it has recently been implemented for surgical treatment [21,22]. However, anatomical parts or medical devices are printed using non-biodegradable polymers such as silicone. Therefore, even if the printed parts conform to the specific anatomies and exert the necessary radial force, clinicians still encounter the usual complications such as mucus plugging or migration, suggesting that congruence of 3D parts is only one of the necessary parameters to consider for prosthesis tolerance [22]. Due to the aforementioned interest in 3D printing for medical devices, the FDA has recently published guidelines [23].

Several biodegradable materials are now available for 3D printing, primarily used in fused deposition modeling (FDM) and stereolithography (STL) technologies.

Polylactic acid (PLA) is the most widely used biodegradable plastic in 3D printing. Derived from renewable resources such as corn starch or sugar cane, it is easy to print with low warpage and is available in various colors and blends. It is suitable for a wide range of applications including prototyping, medical devices, educational projects and consumer products [24]. On the contrary, the variations of PLA such as poly L-lactic acid (PLLA), poly D-lactic acid (PDLA), and poly DL-lactic acid (PDLLA) are less commonly used in 3D printing compared to the standard PLA. Polyhydroxyalkanoates (PHAs) are a family of natural polyesters produced by the bacterial fermentation of sugars or lipids. Derived from renewable sources, it is biocompatible, making it suitable for medical applications, and is widely used in medical devices, packaging and agricultural products [25]. Polycaprolactone (PCL) is a biodegradable polyester with a low melting point. Derived from petrochemical feedstocks but biodegradable, it is flexible and durable. It is used in biomedical applications such as drug delivery systems and tissue engineering [26]. Thermoplastic starch (TPS) blends are derived from crops such as potatoes, corn and wheat. They are, therefore, compostable and are often blended to improve mechanical properties. They can be used for packaging and disposable items [27]. Lignin-based filaments are a by-product of the paper industry and can be used as a component in 3D printing filaments. Derived from woody plants, they are biodegradable and provide good mechanical properties when blended. They are used in combination with other biodegradable polymers for sustainable 3D printing [28]. Gelatin-based material is a natural polymer derived from collagen that can be used for specific 3D printing applications. It is an animal by-product, biocompatible, suitable for bio-printing and used in biomedical applications such as tissue engineering and regenerative medicine [29]. Algae-based filaments are made from algae biomass and offer a sustainable alternative. They are renewable, biodegradable and are mainly used in sustainable product design and prototyping [30]. These biodegradable materials are advancing in quality and variety, driven by the increasing demand for sustainable manufacturing practices. They offer a range of mechanical properties and biodegradability options, making them suitable for different applications from consumer products to medical devices.

While biodegradable materials like PHA, PCL and algae-based filaments have their own unique advantages, PLA stands out for its ease of use, quality of prints, availability and safety. PLA is derived from plant-based materials, making it an environmentally friendly option compared to petroleum-based plastics. It is biodegradable under industrial composting conditions, reducing its environmental impact compared to traditional plastics. PLA has minimal warping and shrinking issues during the printing process and prints at a relatively low temperature (around 180–220 °C), which reduces the risk of printer nozzle clogging and allows for energy savings. Due to its low shrinkage and warping, PLA maintains dimensional accuracy, which is crucial for prototyping and parts that require precise measurements. Finally, PLA is widely available, ensuring competitive pricing and it is compatible with most 3D printers [24].

Three-dimensional printing has been reported in many clinical applications related to airway stenting with controversial results. Guibert et al. [22] used a 3D-printed model of corrected airways to select and customize airway stents, reporting common clinical complications such as mucus plugging and migration. Morrison et al. [31] produced a personalized 3D-printed medical device for the treatment of tracheobronchomalacia. Debiane et al. [32] designed a personalized drug-eluting tracheobronchial stent and quantified tissue changes, such as granulation, associated with airway stenting using stereology. In addition, engineering tools for the design of new tracheobronchial parametric and/or customizable stents have been proposed by Melgoza et al. [33,34], mimicking tracheal physiology; by Xavier Gastal et al. [35], modifying the commercial Dumon silicone prosthesis; by Schopf et al. [36], introducing a new polymeric resorbable stent with a spiral design; and by Zurita et al [37], proposing a fiber-reinforced tubular silicone stent. In addition to the finite element analysis used in these works, other powerful methods can be used for parametric design and subsequent computational analysis, such as the finite difference method [38], the Bezier multistep method [39] and the differential quadrature method [40].

However, to the best of the authors’ knowledge, no systematic analysis has been performed to evaluate the behavior of such a device as a function of geometric variables.

The optimization of biodegradable stents for cardiovascular applications has been carried out by several authors with the aim of evaluating the impact of geometrical features on mechanical performance [41,42,43,44,45,46,47,48,49] or trying to provide optimal designs pursuing specific hemodynamic, structural and geometrical objectives [50,51,52]. Unfortunately, these thresholds are not clearly defined for a tracheobronchial device: the main problem is migration, so radial force should be the criterion for optimization. However, there is a large variability in the type of lesion within patients. They can be benign or malignant, symmetric or asymmetric.

The aim of the study is therefore not to optimize the prosthesis, as there is no clear objective for the optimization process. In fact, it is stated in the literature that the diameter and the length of the prosthesis are selected, as an example, by using a bronchoscope and estimating the radial force in clinics, by the different patients and types of lesions using the difficulty to pass over the stenosis [22]. On the contrary, the present analysis aims to evaluate the importance of selected parameters on the mechanical properties of an airway stent, in particular the radial force, as the stent primarily needs to counteract for the necessary time the force of the stenosis that tends to reduce the airway diameter. Therefore, radial compression tests are performed on several configurations obtained by varying selected parameters of two typical stent patterns that can be printed in 3D. Furthermore, the internal diameter of the stents used in this work was estimated by means of a cadaveric study in rabbits. Thus, the internal diameter is typical of rabbit airways, rather than human airways, because of the possibility of future experimental validation in animals (being pursued in a parallel study). As mentioned above, despite some clinical and experimental trials, no biodegradable device is yet commercially available. The novelty of the present study is twofold: first, it demonstrates the possibility of elucidating the mechanics of a biodegradable stent in terms of its main geometric features using a computational tool based on computer-aided design and finite element methods. Second, the analysis provided is useful for the design of 3D-printable, adaptable and customizable human airway stents to improve the performance of existing endoprostheses and clinical outcomes.

## 2. Materials and Methods

### 2.1. Parametric Stent Model

A diverse array of applications exist for stents, each tailored to specific purposes and featuring distinct geometries. To establish a standardized geometry for parametrization, our study draws inspiration from commercially available metallic stents characterized by X- and W-patterns such as WallStent^©^ (Boston Scientific, Marlborough, MA, USA) and ZilverFlex^©^ stent (Cook Medical, Bloomington, IN, USA), respectively. Despite slight variations between the selected design models, the parameters under scrutiny may not align perfectly due to differences in construction. Consequently, the parametrization efforts are conducted separately for both X and W stent models. The parameters examined in numerical simulations are outlined below; however, it is worth noting that additional parameters exist that, while not considered in the computational study, offer room for customization. For instance, alterations to the inner stent diameter or the prosthesis length permit adaptation to the patient’s trachea and the specific airway lesion at hand.

#### 2.1.1. X-pattern

In this stent model, three design parameters undergo analysis: the pitch angle, the wire thickness, and the number of peaks. Each parameter are scrutinized independently, with the other two parameters are held constant, along with the stent’s final dimensions. All the variations are made from a baseline geometry, characterized by a $60^{{}^{\circ}}$ pitch angle, a strut thickness of 0.4 mm and 11 peaks.

#### Pitch Angle α

**Figure 1:**
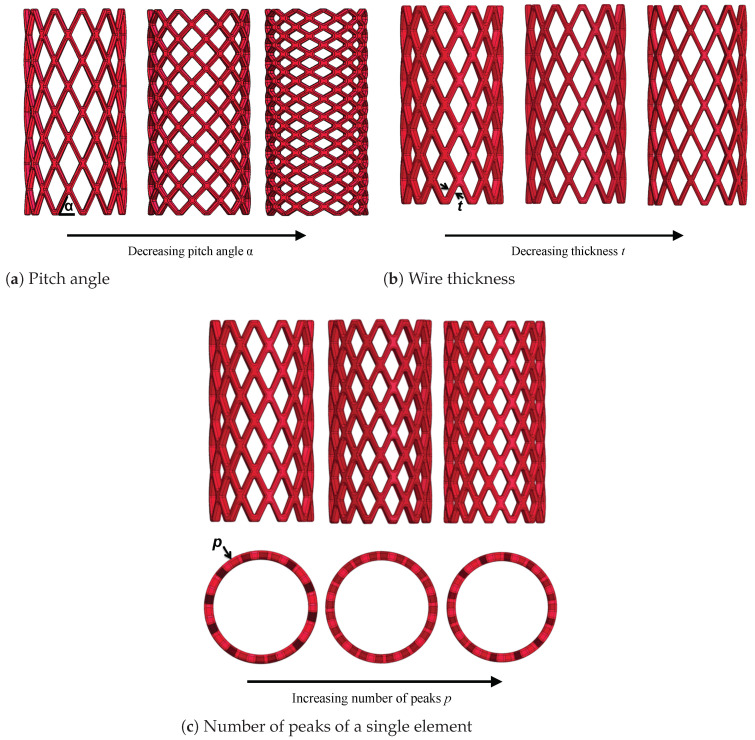
Parameters of the stent model with X-pattern.

The pitch angle, denoted as α and illustrated in Figure 1a, is a critical design parameter. The extensive research highlighted its profound impact on the radial force of the stent [37,48,49,53]. Within this structural context, the pitch angle significantly influences the stent’s radial recoil, thus warranting thorough evaluation in simulations. The values assigned to this parameter also shape the geometry of the cell unit, transitioning from longitudinally to radially oriented rhombuses, with an intermediate squared configuration in between. Specifically, three distinct values are chosen for this parameter, ranging from $60^{{}^{\circ}}$ to $30^{{}^{\circ}}$, with an intermediary value of $45^{{}^{\circ}}$ (refer to Figure 1a).

#### Wire Thickness *t*

This parameter refers to the wire thickness or strut thickness interchangeably. Conventionally, metallic stents have been crafted with thin wires ranging from 0.1 to 0.2 mm. Despite their reduced thickness, they have demonstrated sufficient radial strength owing to the mechanical properties of the metals employed. However, biodegradable polymers typically exhibit weaker characteristics compared to the currently utilized metals in stenting techniques. Consequently, ensuring adequate strength to keep the vessel open and treat lesions while maintaining other design parameters within clinically acceptable ranges becomes challenging [22]. Hence, biodegradable stents are usually thicker than metallic devices. As the strut thickness increases, the contact area between the stent and the vessel wall also increases, which is potentially advantageous for vessel opening. However, it could lead to complications such as heightened interaction with airway tissue. Specifically, with an enlarged contact area, if the stress exerted on the wall is sufficiently high to cause damage, tissue enlargement occurs, prompting cell proliferation in the affected region as an organismic response. This phenomenon may result in airway lumen restriction, known as restenosis. In the present study, thicknesses of 0.4, 0.35 and 0.3 mm are considered (refer to Figure 1b).

#### Number of Peaks (or Cell Units) *p*

Lastly, for this stent model, the number of peaks serves as the final parameter of the design under consideration, with the number transitioning from 11 to 13, with an intermediate value of 12. As depicted in Figure 1c, altering this number impacts the dimensions of the cell unit, which tend to decrease as the number of peaks increases, while maintaining a constant stent diameter. Moreover, this adjustment influences the number of rings in the stent for a constant length. If the dimensions of the cell are preserved in the longitudinal direction, the pitch angle would change. To circumvent this, allowing changes in the number of rings is essential, as we aim to analyze the different parameters separately.

In the Table 1, the analyzed configurations are summarized.

#### 2.1.2. W-pattern

**Figure 2:**
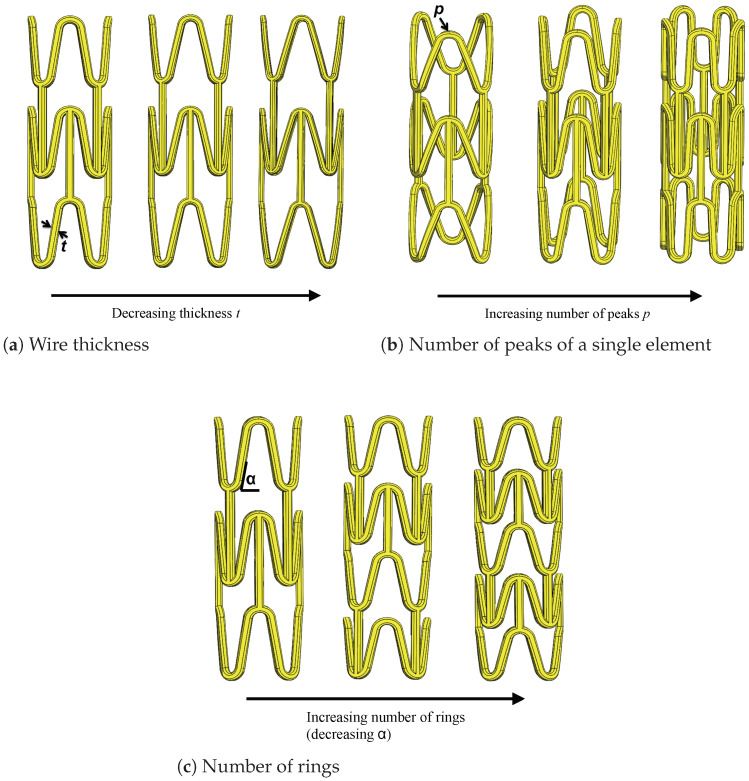
Parameters of the stent model with W-pattern.

Just like the X-pattern, in the W-pattern, three design parameters undergo analysis to study their impact on the stent’s mechanical properties. In this scenario, the three features include the strut thickness, the number of elements (number of rings) and the number of peaks within a single element. The baseline geometry, from which the parameter variations are derived, features a wire diameter of 0.4 mm, 4 peaks and 3 rings (refer to Figure 2a).

#### Wire Thickness *t*

In order to make a direct and clear comparison between the two different models of design, the same three different values for the wire diameter are taken, that is 0.4, 0.35 and 0.3 mm.

#### Number of Peaks (or Cell Units) *p*

To thoroughly analyze the influence of varying the number of peaks on the stent’s performance, the other parameters, along with the stent dimensions, remain constant. Modifying the number of peaks results in a considerable change in the peak-to-peak circumferential distance. This discrepancy becomes evident upon the visual inspection of Figure 2b, while maintaining a constant stent diameter.

#### Number of Rings *r*

In the stents with an X-pattern, the number of rings was altered due to variations in the pitch angle. Conversely, in the W-pattern, studying the variation of the number of rings is simpler than that of the pitch angle. Considering the interdependence between both parameters, their effects on the global geometry seem to be opposite: an increase in the number of rings results in a smaller pitch angle and a denser geometry. Therefore, if the pitch angle is increased, the number of rings decreases, and the geometry becomes less dense. Three different values are considered for the number of rings: 3, 4 and 5, as depicted in Figure 2c.

In Table 2, the analyzed configurations are summarized.

### 2.2. Numerical Models and Boundary Conditions

In accordance with the parametrization outlined earlier, various geometries result from the considered variations for each parameter. In this study, we present the most representative results obtained, totaling 7 configurations for each type of design. These 7 configurations comprise the baseline model and 2 variations for each of the 3 different design parameters established earlier (refer to Table 1 and Table 2).

All stent geometries were designed using SolidWorks (Dassault Systèmes, Simulia, Johnston, RI, USA). Initially, the stents were designed in planar configurations, after which the sketch was wrapped around the external face of a tube with a specific diameter as required. The model was created with editable parameters, facilitating changes and the generation of new configurations. Since SolidWorks is linked with the software package Ansys Mechanical (version 2021 R2, Ansys Inc., Canonsburg, PA, USA) wherein the numerical simulations were conducted, any variation in the geometry of a specific stent is automatically updated in the numerical model. This integration enables the rapid simulation of multiple models, expediting the analysis of various parameters.

**Figure 3:**
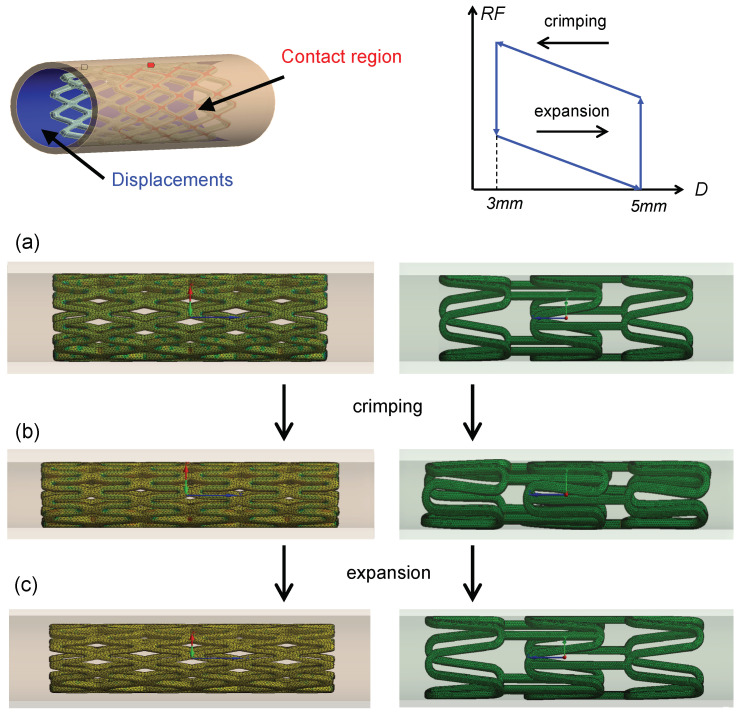
Boundary conditions of the models. The inner cylinder surface initially contacts with the external stent surface. Radial displacements are imposed to the internal cylinder surface. The crimping step is followed by the expansion step. 5 mm and 3 mm are the initial and minimum diameters, respectively. An idealized radial force RF vs. diameter D curve for the radial crimping procedure of a biodegradable stent is shown in the graph. RF values are evaluated in the expansion step. In (**a**–**c**), the situation of the stents before and after crimping and expansion is shown.

**Figure 4:**
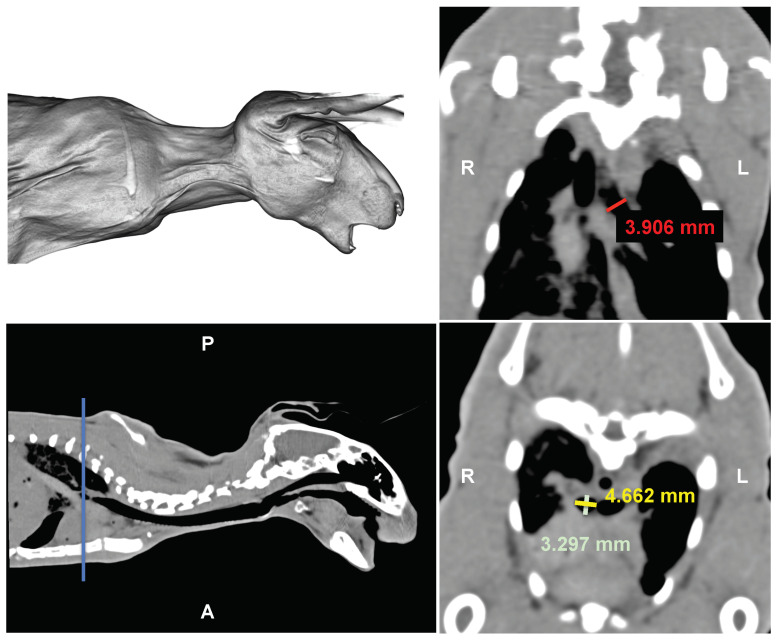
Ex vivo study: Computerized tomography images of a cadaveric rabbit head, neck and thorax. Upper left: 3D rendering. Upper right: Dorsal multiplanar reconstruction. Lower left: sagittal scan. Lower right: transversal scan. Red line: right bronchial length. Yellow and green lines: left bronchial diameters. Blue line: anatomical locator. L = left, R = right, A = anterior, P = posterior.

The crimping head is modeled as a rigid cylinder, with its internal diameter corresponding to the external diameter of the stent (see Figure 3). Radial compression simulation involves applying a radial displacement as a boundary condition. The compression is driven by the cylinder, causing the stent diameter to decrease from its initial value of 5 mm during crimping, to 3 mm during compression and then returning to 5 mm, as shown in Figure 3. This corresponds to a radial deformation of 40%. The initial diameter was selected based on the stent’s testing in rabbits during an ex vivo pilot study (refer to Figure 4). The rationale for using rabbit airway dimensions in the computational study is to assess the initial dimensions of the stents that will be introduced later in the in vivo study in rabbits. This approach aims to evaluate the consequences of the interaction between biological tissue and the medical device. While the in vivo study is being pursued in a parallel work, the objective of the ex vivo study is solely to determine the feasibility of introducing the new stent designs into the rabbit airway, specifically finding the adequate inner radius. As an example, a computerized tomography (CT) scan from a 2.285kg rabbit cadaver is shown in Figure 4. The stents required adaptation to fit the dimensions of the rabbit bronchi, which were measured with the open-source software Horos^TM^ (GNU Lesser General Public License, Version 3 (LGPL-3.0)) revealing a diameter of 3.906, 4.662 and 3.297 mm for the left and the right stem respectively in the absence of airway lesions (see Figure 4). Measurements were obtained separately for the left and right bronchi from the dorsal and transversal thorax scan (see Figure 4 right upper and lower panel). The additional measurements obtained from other rabbits with similar body weights revealed similar measures, in all cases less than 5 mm, which was finally fixed as initial dimension of the device. The interaction between the inner surface of the crimping head and the external surface of the wires was assumed to be frictionless. To prevent rigid body movement, a remote displacement of a node in the middle of each stent was restrained. Finally, the radial force of the stent was computed from the reaction force acting on the cylinder. The simulations were performed using Ansys (release 2020 R2) on an Hp Z2 G4 Workstation equipped with 8 Cores Intel i9, 3.6 GHz and 16 GB RAM.

### 2.3. Material Modeling and 3D Printing

**Figure 5:**
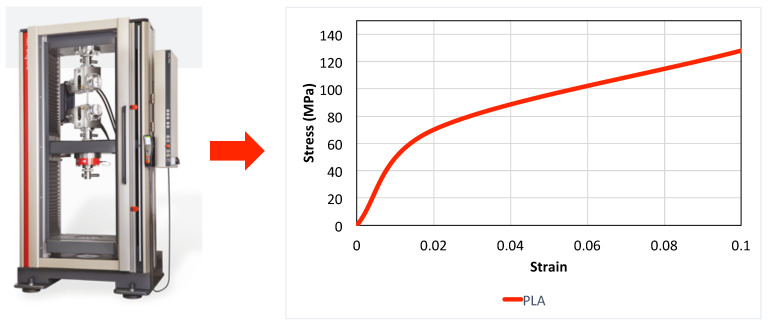
Stress–strain curve model obtained through uniaxial tensile test of a 3D-printed PLA specimen.

For the current study, the biodegradable material chosen for simulations was based on experimental tests conducted at the Asociación de la Industria Navarra (AIN, Pamplona, Spain). We opted for polylactic acid (PLA), a thermoplastic polymer known for its biodegradability properties and its widespread use in 3D printing applications, making it a prominent biomaterial for various medical applications [54]. The initial properties of the PLA used were as follows: density: 1240 kg/m^3^; molecular weight: 20,000 g/mol; degree of crystallinity: 35.4%. A standardized PLA dog bone specimen, prepared according to the ASTM D638 standard procedure [55] and suitable for mechanical testing, underwent a uniaxial tensile test to assess its material properties. For printing the specimen, a 3D printer NX PRO Dual Filament–Filament (TUMaker, Indart 3D, Irún, Spain) was used, with bed temperature: 40–65 °C, printing speed: 50 mm/s and printing temperature: 210 °C). The tensile test was conducted using a Zwick Roell universal testing machine (Zwick GmbH & Co. KG, Ulm, Germany). The following PLA properties were derived from the test: longitudinal elastic modulus E≈3.5 GPa, Poisson’s ratio ν≈0.35, and yield strength ≈70 MPa. The stress–strain curve adapted from the obtained uniaxial tensile test is depicted in Figure 5, alongside the tensile test setup. This curve was implemented in Ansys and utilized for the computational analysis.

### 2.4. Meshing

The stents were meshed using tetrahedral elements, aiming to ensure accurate results. An essential step involved conducting a mesh independence study to determine the optimal element size. Four different element sizes were examined: 0.1, 0.09, 0.085 and 0.08 mm. Notably, no significant changes were observed in the results for the last three meshes analyzed, with changes remaining below 1%. However, the mesh with an element size of 0.1 mm exhibited a notable difference, even below 10%. Consequently, the mesh with an element size of 0.1 mm was deemed inaccurate, while the mesh with an element size of 0.09 mm was selected as the optimal choice. The mesh independence study was conducted for all stent models, considering the various wire diameters.

## 3. Results

### 3.1. X-pattern

The various design parameters for this type of stent model were analyzed independently to discern the influence of each of the stent properties. For the sake of clarity, figures do not display all configurations; rather, only the most representative parameter variations are plotted.

#### 3.1.1. Pitch Angle

**Figure 6:**
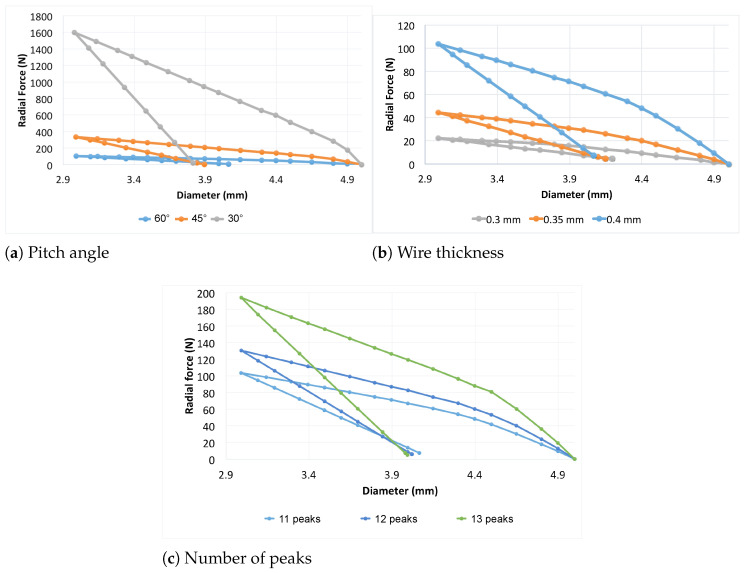
Influence of the pitch angle, wire thickness and number of peaks on the prosthesis designed with X-pattern.

Figure 6a illustrates the variation of the pitch angle α for the specific stent configuration with 11 peaks and a constant strut thickness of 0.4 mm. This trend is consistent across all configurations not displayed. The stent’s radial resistance to compression increases as the pitch angle decreases, regardless of the other parameters. This phenomenon can be attributed to the alteration in the orientation of the cell unit shape, transitioning from a rhombus with its greater diagonal oriented longitudinally to one oriented radially as α decreases. Consequently, the device becomes stiffer. Similar findings have been reported in previous studies [37] and by other authors [48,49]. Additionally, as a consequence of the enhanced radial strength, the radial recoil also increases.

From Figure 6a, it is evident that the maximum radial force exerted by the stent (corresponding to a 40% diameter reduction) decreases drastically from 1600 N to 150 N as the pitch angle α increases from $30^{{}^{\circ}}$ to $60^{{}^{\circ}}$. Furthermore, the figure shows that, in general, the force necessary to compress a stent with $\alpha =30^{{}^{\circ}}$ is higher compared to that required for a stent with $\alpha =30^{{}^{\circ}}$, given a fixed wire thickness. The hysteresis cycle of the gray curve is 17.45 times larger than the blue curve (meaning an increase of 1604.55%) and 4.45 times larger than the orange curve (meaning an increase of 365.78%). This indicates that more energy is needed to compress the stent as the pitch angle decreases.

#### 3.1.2. Wire Thickness

We considered three values for the wire thickness: 0.3, 0.35 and 0.4 mm. As mentioned earlier, the other two parameters were held constant to isolate the effects of changing the strut thickness. Using an example with fixed values for the number of peaks and pitch angle (11 and $60^{{}^{\circ}}$, respectively), Figure 6b illustrates the influence of the wire thickness. Increasing the thickness of the wire results in higher radial forces required to compress the stent to the desired diameter, as expected. Moreover, the increase in thickness also impacts radial recoil, as depicted in the Figure. Thicker wire stents tend toward a smaller diameter after freely expanding from the compressed state. Thus, an increase in strut thickness enhances radial force but leads to greater radial recoil. This is likely due to the reduced gap between the struts for higher thicknesses and early contact between them once the device is compressed. The maximum radial force is achieved at 40% compression for the thicker stent (103.64 N). The 0.35 mm stent has a radial force of 44.58 N, while the 0.3 mm stent has 22.5 N. Thus, increasing the thickness from 0.3 mm to 0.35 mm and then to 0.4 mm increases the radial force by 98.13% and 360.71%, respectively, while increasing from 0.35 mm to 0.4 mm increases the radial force by 132.50%.

#### 3.1.3. Number of Peaks

In all previous comparisons, a fixed number of peaks was maintained. In this analysis, both the strut thickness and the pitch angle remain constant at 0.4 mm and $60^{{}^{\circ}}$, respectively, as an example. The radial strength increases as the number of peaks rises (refer to Figure 6c), as this results in a denser configuration of stent wires. Once again, a greater radial recoil is also evident. The maximum radial force, obtained at a compression of 40% of the devices is obtained for the stent with 13 peaks (194.1 N). This force decreases to 130.56 N for the stent with 12 peaks and to 103.64 N for the stent with 11 peaks. The increase in the number of peaks from 11 to 13 and from 12 to 13 thus results in an increase in the radial force by 87.27% and 48.68%, respectively.

#### 3.1.4. Comparison between Parameters for X-pattern

**Figure 7:**
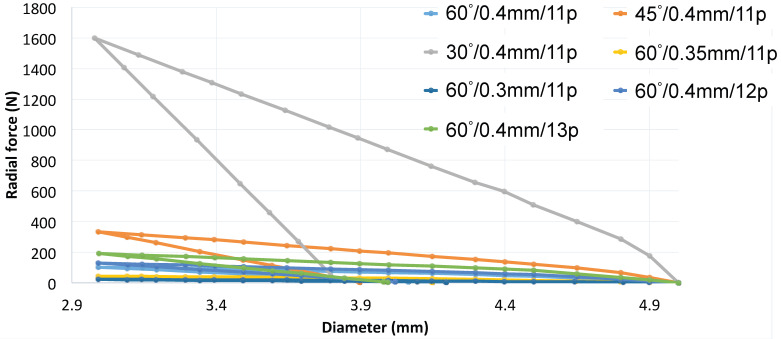
Comparison between different X-pattern configurations.

In Figure 7, the variations of the selected parameters outlined and illustrated above are grouped. For the sake of clarity, given the considerable variability in the force required to compress the prosthesis, as demonstrated in the preceding figures, only a selection of curves is included. These curves are the most representative in terms of radial force versus radial displacements. From this comprehensive comparison, it becomes apparent that the variation adopted for the pitch angle exerts the most significant influence on the stent behavior.

### 3.2. W-pattern

As previously carried out with the X-pattern, the different design parameters for the stent with the W-pattern were initially analyzed independently to discern the influence each has on the stent’s properties. Specifically, the radial force of the stent was studied by simulating a compression test for each of the different configurations. Subsequently, a global comparison is presented.

#### 3.2.1. Wire Thickness

**Figure 8:**
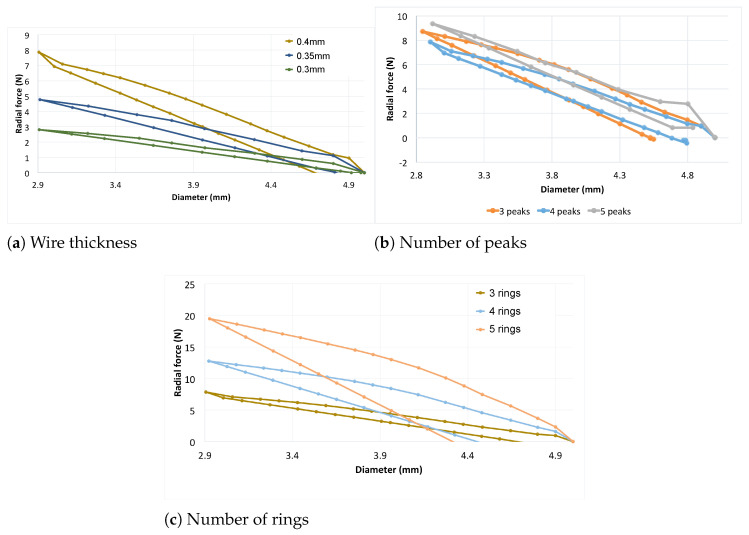
Influence of the wire thickness, number of peaks and number of rings on the prosthesis designed with W-pattern.

Here, the influence of the wire thickness is compared for configurations having three rings and four peaks. In this scenario, the radial strength tends to increase as the wire diameter is increased (refer to Figure 8a). However, due to the particular design, this type of stent model appears to be considerably more flexible than the X-pattern. Given that the radial force is much lower than that of X-pattern models, the amount of plastic strain is notably lower, as evidenced by the larger diameter after expansion. Consequently, the radial recoil is also very low when the stent is free to expand. Thus, in this case, increasing the wire diameter promotes a stiffer stent with no significant radial recoil. Additionally, the paths followed by the loading and unloading processes are similar, at least with similar slopes, which differs from the hysteresis observed in models with X-patterns. The maximum radial force is achieved at 40% compression for the thicker stent (7.86 N). The 0.35 mm stent has a radial force of 4.77 N, while the 0.3 mm stent has 2.81 N. Thus, increasing the thickness from 0.3 mm to 0.35 mm and then to 0.4 mm increases the radial force by 69.75% and 179.64%, respectively, while increasing from 0.35 mm to 0.4 mm increases the radial force by 64.66%.

#### 3.2.2. Number of Peaks

Here, the influence of the number of peaks on radial thickness is compared using three rings and a thickness of 0.4 mm. As mentioned, this parameter directly affects the pitch angle of a single ring. The number of peaks radically changes the geometry of the device and is expected to influence its mechanical performance. Although the number of peaks increases, the radial strength varies without a clear tendency, as observed in Figure 8b. An increase in force can be seen from the four peak to five peak model. However, an increase is also visible when the number of peaks decreases from four to three, particularly at large crimping diameters. Additionally, the model with three peaks exhibits significant hysteresis; due to its geometry, a higher force is needed to crimp the stent compared to the other two models, and this also influences the recoil. The maximum radial force, obtained at a compression of 40% of the devices, is obtained for the stent with five peaks (9.36 N). This force decreases to 8.71 N for the stent with three peaks and to 7.86 N for the stent with four peaks. The change in the number of peaks from three to four, from four to five and from three to five thus results in variations in the radial force of 10.81%, 19.08% and 7.46%, respectively.

#### 3.2.3. Number of Rings

Here, the number of peaks and the wire thickness are maintained constant at four rings and 4 mm, respectively. We have examined configurations with three, four and five rings. The trend followed by the radial strength is clearly depicted in Figure 8c. As the number of rings for the same length increases, the structure of the stent becomes denser, resulting in increased radial force. For the highest number of rings analyzed here, which is five, the yield limit is reached at some point in the stent, leading to radial recoil. The maximum force at minimum diameter decreases from 19.2 N to 12.9 N and 6.7 N, indicating again that if the pitch angle increases (number of rings decreases for W-pattern), the radial force necessary to compress the stent deceases. In this case, the area under the hysteresis cycles of the orange curve is 417.86% larger than the brown and 95.29% larger than the blue one.

#### 3.2.4. Comparison between Parameters for W-pattern

**Figure 9:**
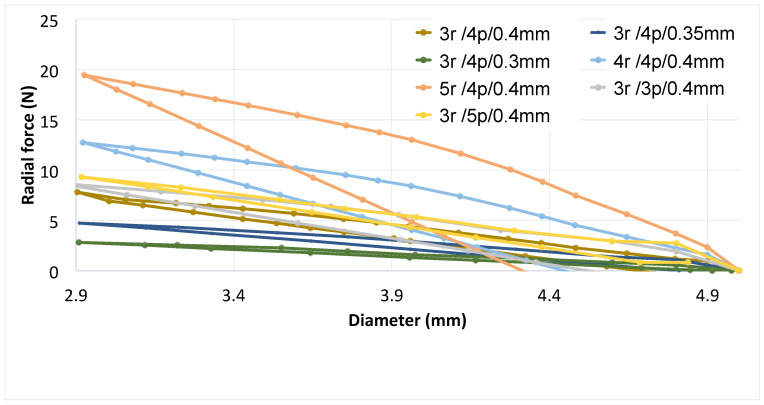
Comparison between selected parameter variations on the radial force of the W designed prosthesis.

From the comparison of some of the analyzed parameters in the W-designed prosthesis (Figure 9), it appears that the most rigid prosthesis is the one with the highest number of peaks, while the least rigid is the one with the lowest strut thickness, regardless of the configuration.

#### 3.2.5. Comparison between X- and W-pattern

**Figure 10:**
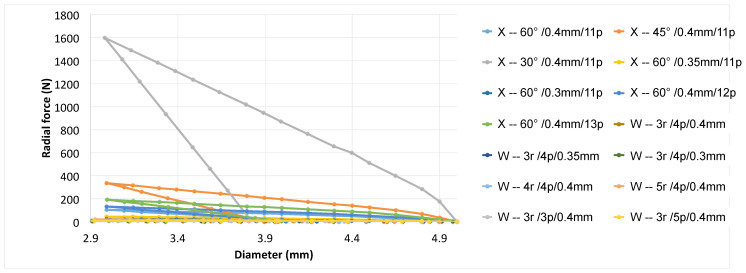
Comparison between selected X- and W-patterns.

From Figure 10, it is evident that prostheses with the X-pattern exhibit greater radial force compared to those with the W-pattern. This can be attributed to the denser structure of the X-pattern, resulting in fewer gaps and less space for displacement driven by the analyzed parameters, especially the pitch angle. In terms of radial recoil, the devices designed with the X-pattern did not expand to their original diameter, whereas for stents in the W-pattern, the recoil is markedly smaller, allowing them to recover a large part of their initial configuration. This is explained by geometric features such as the lack of space for displacement in the X-pattern stent, causing the struts to join as a radial spring under radial compression and leading to higher stresses that exceed the yield limit. Since plastic strain contributes to radial recoil, achieving full expansion of the stent requires applying greater force to it. However, excluding the curves represented in gray (X/$30^{{}^{\circ}}$/0.4 mm/11p) and orange (X/$45^{{}^{\circ}}$/0.4 mm/11p), corresponding to the pitch angles $\alpha =30^{{}^{\circ}}$ and $\alpha =45^{{}^{\circ}}$, it is evident that devices designed in W-patterns and X-patterns exhibit very similar radial force. Particularly, stents designed with a W-pattern and thickness of 0.35 or 0.4 mm show higher radial force compared to those with the X-pattern at high pitch angles (see Figure 10). This demonstrates that even though the X-pattern configuration is visibly stiffer than the W-pattern, manipulating the parameters allows for creating devices with the same force starting from different designs.

## 4. Discussion

The use of tracheobronchial stents for the treatment of human airway pathologies is still inefficient and controversial due to contraindications. Common side effects still lead to reintervention [22]. Plastic prostheses are particularly affected by migration and obstruction [9]. Metal stents are thinner and therefore more flexible. Nevertheless, these devices facilitate a strong tissue reaction with abnormal cell proliferation, inflammation and granuloma formation, as reported in the literature [56]. Silicone prostheses have been subject to improvements in the last decades [33,35,37,57,58] without clear benefits. Furthermore, metallic stents can be easily inserted by flexible bronchoscopy and conscious sedation [4], whereas silicone stents require rigid bronchoscopy and general anesthesia [9]. Biodegradable stents are theoretically promising because they combine the advantages of metallic and plastic stents in terms of ease of insertion and stability but are programmed to disappear before the side effects affect the patient. However, no biodegradable airway stents are currently commercially available. Trials have been carried out in adults [59] and children [60]. These prostheses were used to treat transient airway stenoses and four out of six adult patients and both treated children required further intervention and re-stenting, suggesting too rapid degradation. Therefore, although this concept is attractive, more work is needed and more knowledge is required before biodegradable stents could be considered a concrete alternative to silicone, metallic or hybrid stents [61]. In particular, specific properties such as radial force and clinical complications of biodegradable stents need to be compared with those of conventional stents before being adopted in the clinic, and further studies on the materials are needed to assess the degradation time required for each specific patient condition [22]. Biodegradable stents have also been tested in rabbits with the precise aim of analyzing the biodegradability of specific materials in the respiratory tract [36,62,63]. All of these studies found good biocompatibility between the device and the airway tissue and suggest further investigation into degradation time and stent structure.

From this picture, it is clear that knowledge is required to produce a biodegradable stent that could solve the contraindication without losing efficiency. Therefore, in this work we have developed a numerical methodology based on finite element modeling to analyze the influence of the key design parameters of two 3D printable airway stents on their mechanical behavior, in order to try to predict their performance in terms of personalization to the patient and the lesion. The radial force of the prostheses depends mainly on their thickness, so that since the modulus of elasticity of silicone is much lower than that of metals, silicone prostheses are thicker with respect to metallic stents [35]. Since the FDA indicates the use of metallic stents only when the pathology cannot be treated by other means, and covered metallic stents are actually recommended to avoid long-term re-stenosis, 3D printing biodegradable uncovered stents offers a new valuable solution. Firstly, the device can be customized to the patient (diameter and length) and to the type of lesion (radial force, thickness, design). Secondly, as mentioned above, since the material degrades over time, the airway can only be supported for the necessary time without causing unwanted side effects. In this work, we have studied the feasibility of a customizable airway stent, evaluating the mechanical properties as a function of strut thickness, controlled by the 3D printer and the stent pattern. The W-pattern is generally less stiff than the X-pattern. However, we have shown that by manipulating the parameters, it is possible to create different radial forces using the two designs.

FEM studies in the literature have shown that polymers can be used to design biodegradable cardiovascular stents. Mehdi-Torki et al. [51] demonstrated that PLA-based stents can be properly used to open atherosclerotic arteries and provide the necessary force if proper stent design and optimization are applied. Pauck et al. [64] have investigated the effect of geometry and material properties on the performance of a PLLA stent for cardiovascular applications. Through a series of tests, they evaluated the mechanical properties of the stent as a function of device geometry, design characteristics, radial force and radial strength. The effect of design parameters on the mechanical properties of different polyester, polyamide and polypropylene stents has been analyzed by Rebelo et al. [53]. They systematically estimated the influence of fiber filament type, braiding angle and mandrel diameter using experimental compression and bending tests. These studies demonstrated the current interest in biodegradable materials for stent applications, even in the cardiovascular field, where the FDA already accepts the treatment of atherosclerotic lesions with such devices. In contrast, little is known about stenting in the airways, and much less work has been carried out. To the authors’ knowledge, no systematic work has attempted a comprehensive analysis of biodegradable airway devices. This work is a first step in this direction.

### Limitations of the Study

The main limitations of the present study include the lack of experimental results on the interaction between stent and tissue and the biodegradation time of PLA in the selected designs. With the computational analysis, it is possible to simulate the interaction between the prosthesis and the biological tissue [65,66]. The latter could, in fact, assess the locations of higher stresses during the physiological maneuvers, giving an indication of where tissue inflammation and granulation may occur. This task will be left to future studies. In addition, an experimental study of degradation time needs to be carried out to assess the correct time of degradation and, as a consequence, possible foreign body reaction after implantation or possible re-epithalization, indicating a rapid biological response. While this study will be continued in a parallel work, in the present study, we have focused on the mechanical properties and the ability to design customizable devices with the aim of generating a useful tool for prosthesis design, customization and analysis. In addition, the study neglects the simulation of biodegradation processes and their effect on mechanical stability over time. Stents are considered in their initial conditions in terms of material integrity. However, it is well known that loss of integrity leads to loss of mechanical properties. The consequences of this aspect have not been taken into account in the simulations presented. Furthermore, as mentioned above, optimization of the prosthesis was not carried out and is not the aim of the study. Although important for implantation in a specific patient, this aspect is particularly challenging in the respiratory field due to the variability of lesion types and airway geometries. For example, for cardiovascular biomechanics, a categorization of patients and pathologies has been proposed in the literature [67]. However, a clear objective of such optimization is not defined for airway lesions, and usually, clinicians select the length and size of the airway stent by experience [22]. Therefore, the present study is limited to the influence of design parameters on the mechanical properties of the stent. In general, a non-degradable prosthesis is considered a foreign body and, as such, is susceptible to promote side effects. The present study, with all its limitations, is therefore an attempt to help the stenting technique to better understand the use of biodegradable materials before they are used in clinics.

## 5. Conclusions

In this study we analyzed the effect of several key design parameters on the mechanical properties of a biodegradable airway stent. The properties of biodegradable stents are often poor due to the low radial force of the device. Treatment of tracheal stenosis is still problematic, and stent implantation usually requires repeat surgery due to various side effects. The biodegradable devices with appropriate properties and designs would offer an alternative solution with the advantage of reducing the drawbacks. The results of the proposed simulations confirm that there is a direct correlation between stent design and mechanical properties through stent thickness and pitch angle. In addition, a stent designed with an X-pattern was generally stiffer than a stent designed with a W-pattern. However, by manipulating the parameters, the two designs can significantly change the radial behavior and even achieve similar radial force. Since the placement of airway stents is determined by a large variability of clinical factors, and their radial force can vary greatly depending on the type of lesion and the patient, the framework presented in this work shows that the possibility of selecting and fabricating a specific device for a specific patient and lesion can, at least theoretically, be extended. The present work offers a methodology that, through computer-aided design and numerical calculations, can provide a customizable stent that can be numerically analyzed and printed in 3D. This numerical tool is fully automated and allows the analysis of a fixed configuration as a function of several parameters or, conversely, the analysis of several designs with fixed parameters. This study could potentially be useful for further analysis of the design, development and optimization of biodegradable polymer-based airway stents, helping to address the failure of currently existing stents.

## Figures and Tables

**Table 1:** Considered parameters and print volume for different configurations of airway stents with the X-pattern.

Parameters	Model #1	Model #2	Model #3	Model #4	Model #5	Model #6	Model #7
Thickness *t* (mm)	0.4	0.35	0.3	0.4	0.4	0.4	0.4
Number of peaks *p*	11	11	11	11	11	12	13
Pitch angle α (°)	60	60	60	45	30	60	60
Volume (mm^3^)	35.07	24.84	21.36	39.78	49.71	37.25	39.12

**Table 2:** Considered parameters and print volume for different configurations of airway stents with W-pattern.

Parameters	Model #1	Model #2	Model #3	Model #4	Model #5	Model #6	Model #7
Thickness *t* (mm)	0.4	0.35	0.3	0.4	0.4	0.4	0.4
Number of peaks *p*	4	4	4	4	4	3	5
Number of rings *r*	3	3	3	4	5	3	3
Volume (mm^3^)	25	18.53	13.22	27.37	29.76	19.15	31.08

## Data Availability

The raw data supporting the conclusions of this article will be made available by the authors on request.
